# Supplementary material for: How Do Neighbourhood Definitions Influence the Associations between Built Environment and Physical Activity?
Source: Int J Environ Res Public Health. 2019 Apr 28;16(9):1501. doi: 10.3390/ijerph16091501 (PMC6540146; doi:10.3390/ijerph16091501)
Supplement: Supplementary file 1 [file ijerph-16-01501-s001.pdf]

Supplementary tables

Table S1. Results from the fully adjusted models where the outcome is accelerometer counts. All models are fully adjusted for sex, age, ethnicity, income, marital status, education, employment, car access, neighbourhood socioeconomic deprivation, and neighbourhood preference.

Built environment measure	Neighbourhood definition	Coefficient	Standard error	95% Confidence interval	
Dwelling density	MB	0.0063	0.0013	0.0037	0.0089
Dwelling density	CA	0.0086	0.0032	0.0024	0.0149
Dwelling density	UN	0.0082	0.0020	0.0043	0.0120
Dwelling density	B0500	0.0105	0.0026	0.0055	0.0155
Dwelling density	B0800	0.0115	0.0032	0.0053	0.0177
Dwelling density	B1000	0.0116	0.0036	0.0046	0.0187
Dwelling density	B1500	0.0118	0.0040	0.0039	0.0196
Street connectivity	MB	0.0002	0.0003	-0.0005	0.0008
Street connectivity	CA	0.0037	0.0013	0.0011	0.0063
Street connectivity	UN	0.0027	0.0007	0.0015	0.0040
Street connectivity	B0500	0.0028	0.0010	0.0009	0.0047
Street connectivity	B0800	0.0037	0.0012	0.0014	0.0061
Street connectivity	B1000	0.0045	0.0013	0.0020	0.0071
Street connectivity	B1500	0.0039	0.0013	0.0013	0.0065
NDAI	MB	-0.0100	0.0053	-0.0205	0.0005
NDAI	CA	0.0088	0.0034	0.0021	0.0155
NDAI	UN	-0.0017	0.0058	-0.0132	0.0097
NDAI	B0500	0.0052	0.0032	-0.0010	0.0114
NDAI	B0800	0.0080	0.0024	0.0033	0.0128
NDAI	B1000	0.0063	0.0021	0.0021	0.0105
NDAI	B1500	0.0059	0.0019	0.0021	0.0097

Table S2. Results from the fully adjusted models where the outcome is percentage time in MVPA. All models are fully adjusted for sex, age, ethnicity, income, marital status, education, employment, car access, neighbourhood socioeconomic deprivation, and neighbourhood preference.

Built environment measure	Neighbourhood definition	Coefficient	Standard error	95% Confidence interval	
Dwelling density	MB	0.0063	0.0013	0.0007	0.0086
Dwelling density	CA	0.0086	0.0032	0.0020	0.0219
Dwelling density	UN	0.0082	0.0020	-0.0005	0.0136
Dwelling density	B0500	0.0105	0.0026	0.0015	0.0180
Dwelling density	B0800	0.0115	0.0032	0.0002	0.0209
Dwelling density	B1000	0.0116	0.0036	-0.0033	0.0197
Dwelling density	B1500	0.0118	0.0040	-0.0076	0.0182
Street connectivity	MB	0.0002	0.0003	-0.0009	0.0007
Street connectivity	CA	0.0037	0.0013	0.0017	0.0096
Street connectivity	UN	0.0027	0.0007	0.0005	0.0053
Street connectivity	B0500	0.0028	0.0010	0.0000	0.0055

Street connectivity	B0800	0.0037	0.0012	0.0008	0.0075
Street connectivity	B1000	0.0045	0.0013	0.0005	0.0081
Street connectivity	B1500	0.0039	0.0013	-0.0018	0.0063
NDAI	MB	-0.0100	0.0053	-0.0218	0.0043
NDAI	CA	0.0088	0.0034	-0.0067	0.0149
NDAI	UN	-0.0017	0.0058	-0.0080	0.0314
NDAI	B0500	0.0052	0.0032	-0.0063	0.0097
NDAI	B0800	0.0080	0.0024	0.0015	0.0149
NDAI	B1000	0.0063	0.0021	0.0004	0.0123
NDAI	B1500	0.0059	0.0019	-0.0006	0.0107

Table S3. Results from the fully adjusted models where the outcome is self reported walking for transport (time in minutes). All models are fully adjusted for sex, age, ethnicity, income, marital status, education, employment, car access, neighbourhood socioeconomic deprivation, and neighbourhood preference.

**Built environment measure**	**Neighbourhood definition**	**Coefficient**	**Standard error**	**95% Confidence interval**	
Dwelling density	MB	0.0225	0.0076	0.0076	0.0373
Dwelling density	CA	0.0485	0.0169	0.0153	0.0816
Dwelling density	UN	0.0458	0.0121	0.0221	0.0695
Dwelling density	B0500	0.0488	0.0151	0.0192	0.0783
Dwelling density	B0800	0.0512	0.0191	0.0138	0.0886
Dwelling density	B1000	0.0519	0.0217	0.0094	0.0945
Dwelling density	B1500	0.0653	0.0246	0.0171	0.1136
Street connectivity	MB	0.0018	0.0016	-0.0014	0.0051
Street connectivity	CA	0.0213	0.0071	0.0074	0.0352
Street connectivity	UN	0.0134	0.0038	0.0059	0.0209
Street connectivity	B0500	0.0094	0.0052	-0.0007	0.0195
Street connectivity	B0800	0.0056	0.0066	-0.0073	0.0185
Street connectivity	B1000	0.0150	0.0074	0.0005	0.0294
Street connectivity	B1500	0.0275	0.0076	0.0125	0.0424
NDAI	MB	-0.0157	0.0266	-0.0678	0.0363
NDAI	CA	0.0464	0.0190	0.0091	0.0837
NDAI	UN	-0.0361	0.0329	-0.1006	0.0283
NDAI	B0500	0.0113	0.0161	-0.0202	0.0429
NDAI	B0800	0.0351	0.0126	0.0104	0.0598
NDAI	B1000	0.0390	0.0109	0.0176	0.0605
NDAI	B1500	0.0280	0.0103	0.0077	0.0482

Table S4. Results from the fully adjusted models where the outcome is self reported walking for recreation (time in minutes). All models are fully adjusted for sex, age, ethnicity, income, marital status, education, employment, car access, neighbourhood socioeconomic deprivation, and neighbourhood preference.

**Built environment measure**	**Neighbourhood definition**	**Coefficient**	**Standard error**	**95% Confidence interval**	
Dwelling density	MB	0.0310	0.0076	0.0161	0.0459

Dwelling density	CA	0.0311	0.0177	-0.0036	0.0657
Dwelling density	UN	0.0439	0.0115	0.0213	0.0664
Dwelling density	B0500	0.0425	0.0150	0.0131	0.0719
Dwelling density	B0800	0.0559	0.0184	0.0198	0.0920
Dwelling density	B1000	0.0669	0.0208	0.0261	0.1076
Dwelling density	B1500	0.0748	0.0236	0.0286	0.1210
Street connectivity	MB	0.0005	0.0018	-0.0030	0.0040
Street connectivity	CA	0.0149	0.0074	0.0003	0.0295
Street connectivity	UN	0.0116	0.0039	0.0039	0.0193
Street connectivity	B0500	0.0090	0.0054	-0.0017	0.0196
Street connectivity	B0800	0.0208	0.0067	0.0077	0.0338
Street connectivity	B1000	0.0207	0.0074	0.0062	0.0353
Street connectivity	B1500	0.0279	0.0073	0.0135	0.0422
NDAI	MB	0.0118	0.0287	-0.0443	0.0680
NDAI	CA	0.0389	0.0194	0.0009	0.0770
NDAI	UN	0.0107	0.0329	-0.0538	0.0752
NDAI	B0500	0.0092	0.0173	-0.0247	0.0430
NDAI	B0800	0.0139	0.0137	-0.0129	0.0407
NDAI	B1000	0.0175	0.0120	-0.0061	0.0411
NDAI	B1500	0.0096	0.0112	-0.0124	0.0316

Table S5. Results from the fully adjusted models where the outcome is self reported overall walking (time in minutes). All models are fully adjusted for sex, age, ethnicity, income, marital status, education, employment, car access, neighbourhood socioeconomic deprivation, and neighbourhood preference.

Built environment measure	Neighbourhood definition	Coefficient	Standard error	95% Confidence interval	
Dwelling density	MB	0.0192	0.0068	0.0060	0.0325
Dwelling density	CA	0.0181	0.0159	-0.0131	0.0492
Dwelling density	UN	0.0407	0.0104	0.0202	0.0611
Dwelling density	B0500	0.0393	0.0136	0.0127	0.0659
Dwelling density	B0800	0.0465	0.0170	0.0131	0.0799
Dwelling density	B1000	0.0480	0.0193	0.0101	0.0859
Dwelling density	B1500	0.0575	0.0220	0.0144	0.1006
Street connectivity	MB	0.0010	0.0015	-0.0019	0.0039
Street connectivity	CA	0.0149	0.0065	0.0020	0.0277
Street connectivity	UN	0.0104	0.0034	0.0037	0.0171
Street connectivity	B0500	0.0099	0.0046	0.0007	0.0190
Street connectivity	B0800	0.0122	0.0059	0.0007	0.0237
Street connectivity	B1000	0.0140	0.0066	0.0010	0.0270
Street connectivity	B1500	0.0153	0.0071	0.0014	0.0293
NDAI	MB	0.0005	0.0242	-0.0469	0.0479
NDAI	CA	0.0490	0.0164	0.0168	0.0813
NDAI	UN	0.0115	0.0292	-0.0456	0.0687
NDAI	B0500	0.0336	0.0144	0.0053	0.0619
NDAI	B0800	0.0401	0.0112	0.0182	0.0620

NDAI	B1000	0.0408	0.0097	0.0218	0.0597
NDAI	B1500	0.0278	0.0092	0.0098	0.0458
